# Association Between Donor HLA-DQ Heterodimers Group and Graft Loss in Kidney Transplant Recipients

**DOI:** 10.1097/TXD.0000000000001998

**Published:** 2026-09-09

**Authors:** Marie-Pier Thivierge, Olivier Désy, Lara Aguiar, Eric Wagner, Eva Latulippe, Julie Riopel, Sacha A. De Serres

**Affiliations:** 1 Transplantation Unit, Renal Division, Department of Medicine, CHU de Québec, Faculty of Medicine, Université Laval, Québec, QC, Canada.; 2 Immunology and Histocompatibility Laboratory, CHU de Québec, Faculty of Medicine, Université Laval, Québec, QC, Canada.; 3 Department of Laboratory Medicine, CHU de Québec, Faculty of Medicine, Université Laval, Québec, QC, Canada.

## Abstract

**Background.:**

Combinations of HLA-DQ α1 and β1 chains are restricted to specific groups: group 1 (G1), comprising DQA1*02/03/04/05/06 with DQB1*02/03/04, and group 2 (G2), comprising DQA1*01 with DQB1*05/06. A recently discovered role of HLA-DQ heterodimers in hematopoietic cell transplantation suggests poorer outcomes in patients carrying G2 heterodimers. However, the absence of a clear biological rationale has raised questions regarding the generalizability of this observation.

**Methods.:**

To further evaluate this finding, we investigated the impact of the HLA-DQ group genotype in 821 kidney recipients by multivariate survival modeling.

**Results.:**

Compared with recipients of G1G1 donor grafts, those who received G2G2 donor grafts had a 1.9-fold (95% confidence interval [CI], 1.2-2.9; *P* < 0.01) increased risk of graft loss, independent of HLA-DQ and HLA-DR donor-recipient mismatches. Analysis of graft losses due to biopsy-proven rejection showed that this risk increased with an adjusted hazard ratio of 4.3 (95% CI, 1.1-16.4; *P* = 0.03). When further analyzed according to DQB1 alleles within the G1 genotype, these associations were restricted to HLA-DQB1*03 alleles, corresponding to DQ7, DQ8, and DQ9. Compared with recipients of donors possessing 2 HLA-DQB1*03 alleles, those with no DQB1*03 but 2 DQB1*05/06 alleles exhibited a 2.5-fold (95% CI, 1.3-4.5; *P* = 0.01) increased risk of graft failure, suggesting a biological gradient of effect. In sensitivity analyses, these results were independent of the usual predictors of graft loss.

**Conclusions.:**

The paradigm based on HLA-DQ heterodimers appears to be shared between hematopoietic and solid organ transplantation, strongly encouraging investigation into the underlying biological mechanisms.

## INTRODUCTION

The HLA complex presents antigens derived from self and non-self-sources to T cells. Class II HLA molecules are heterodimers composed of α and β chains and are divided into 3 main subclasses: HLA-DR, HLA-DQ, and HLA-DP. HLA-DP and HLA-DQ both exhibit polymorphism in the α1 and β1 chains, whereas HLA-DR displays polymorphism in the β1 chain, with very limited polymorphism in the α1 chain.^1^ For reasons that are not yet fully understood, combinations of DQ α and β chains are restricted to specific groups. Group 1 (G1), comprising α chains encoded by DQA1*02, *03, *04, *05, or *06, forms noncovalent associations only with β chains encoded by DQB1*02, *03, or *04. In contrast, group 2 (G2), comprising α chains encoded by DQA1*01, combines only with β chains encoded by DQB1*05 or *06.^2^ Using gene transfer with retroviral vectors, Kwok et al^3^ demonstrated that DQβ chains of G2 do not form stable heterodimers with DQα chains of G1, and vice versa. This biological characteristic implies that the diversity of HLA-DQ heterodimers expressed on the human cell surface depends on the individual’s G1 and G2 genotypes. For instance, individuals homozygous for HLA-DQ express a single heterodimer on the cell surface, whereas G1G2 individuals express 2 unique heterodimers. In contrast, G1G1 and G2G2 individuals can generate additional α-β combinations through transdimerization, resulting in the expression of up to 4 distinct HLA-DQ heterodimers.^2,4^

The clinical impact of HLA-DQ heterodimer matching has been investigated recently in hematopoietic cell transplantation. Petersdorf et al^5^ reported that G2 molecules are associated with a higher risk of relapse compared with G1 molecules. Importantly, this observation was made in HLA-matched recipients, demonstrating that it was not attributable to HLA alloreactivity. Moreover, G2 molecules were predictive of poorer disease-free survival in both matched and mismatched individuals, suggesting a detrimental effect. As stated in an accompanying editorial, these findings are somewhat unexpected and currently lack a clear biological rationale.^6^ Therefore, demonstrating consistent results in solid organ transplantation would strengthen the robustness of these observations.

In relation to these findings, we recently reported that, in human endothelial cells with naturally occurring HLA complexes, the surface protein levels of HLA-DQ molecules were higher in HLA-DQB1*05 and -DQB1*06 positive individuals (G2) than in those with HLA-DQB1*03 (G1).^7^ Consistent with these in vitro data, we observed a significant increase in microvascular inflammation on graft biopsies in 2 cohorts of kidney transplant recipients matched for HLA-DR antigens but mismatched for HLA-DQB1*05 or -DQB1*06.^7^ To date, the influence of G1/G2 genotypes on kidney transplantation outcomes has been little investigated, has mainly been assessed in the context of classical HLA-DQ mismatching, or has focused analyses on the impact of G2 matching.^8-10^

This study aimed to evaluate the association between donor G1 and G2 molecules and graft loss following kidney transplantation. Given the observation that HLA-DQ protein levels were higher in patients with the G2 phenotype, we hypothesized that donor G2 molecules may be associated with increased immune recognition and exert a deleterious effect on outcomes compared with G1 molecules.

## MATERIALS AND METHODS

### Study Design and Population

This observational cohort study included all 821 kidney transplant recipient-donor pairs transplanted at the University Health Center of Québec between January 3, 2000, and February 9, 2024, for whom HLA-DQB1 genotyping data were available. There were no patients with preformed donor-specific antibodies (DSAs) in the cohort.

### Outcomes

The primary outcome was graft failure, defined as either death irrespective of graft function or a return to dialysis. The secondary outcomes were graft loss due to biopsy-proven rejection (identified as the primary cause by chart review) and death-censored graft failure. Survival time was calculated from the date of transplantation. The censoring criterion was the absence of the specified endpoint at the last follow-up. This last follow-up corresponded to the date of the last visit to the kidney transplant clinic, whether in person or via telemedicine.

### HLA Genotyping

Recipients and donors were HLA-typed at the Clinical Histocompatibility Laboratory in accordance with approved clinical protocols. Before 2009, patient and donor HLA-A, B, C, DRB1, DRB3/4/5, DQB1 typing was performed at low resolution using sequence-specific primer typing kits (One Lambda, West Hills, CA). From 2009 on, HLA-A, B, C, DRB1, DRB3/4/5, DQA1, DQB1, DPA1, and DPB1 typing was performed at low- to intermediate-resolution using a Luminex-based sequence-specific oligonucleotide typing kit (One Lambda, or Immucor, Waukesha, WI). In cases of ambiguity, typing was repeated using intermediate- to high-resolution sequence-specific primer kits (One Lambda).

### Histological Assessment

Biopsies were prospectively graded by local attending renal pathologists (E.L. and J.R.) according to the Banff 1997 criteria and their subsequent updates.^11-18^ All biopsies were reviewed by a complete team of pathologists and nephrologists during weekly clinicopathological transplant conferences, where challenging cases were discussed. Since 2013, a protocol follow-up biopsy has been offered to all patients at 6 and 24 mo posttransplant. Patient acceptance varies depending on several factors, including antiplatelet therapy, the distance between home and hospital (due to the logistics of returning home after an outpatient biopsy), and renal function. The standard biopsy was also omitted at the clinician’s discretion when a recent biopsy had been performed for other indications. In total, 1105 biopsies were performed in 558 patients (68% of the total cohort) during the follow-up period.

### Anti-HLA Antibody Assessment

Serum samples were screened for de novo DSA (dnDSA) by flow cytometry using FlowPRA beads (One Lambda, Canoga Park, CA). Between 2005 and 2012, identification was performed using single-flow antigen beads. Since 2012, the Luminex Platform has been used to identify HLA antibodies using LABScreen single-antigen beads (One Lambda). A normalized mean fluorescence intensity cutoff value of ≥1500 was used to detect dnDSA, and their presence was confirmed in each case by the HLA laboratory director (E.W.) via eplet analysis. When DQA1 typing was unknown, dnDSA determination was performed by association analysis based on the most frequent and published DRB1-DQB1-DQA1 haplotypes (E.W.). Surveillance for dnDSA was performed at months 1, 3, 6, and 12 posttransplant and yearly thereafter; screening for dnDSA was also performed at the time of any graft biopsy.

### Study Approval

The Institutional Ethics Committee of the University Hospital (Project 2024-6949) approved this study. The requirement for informed consent was waived by the committee. All clinical and research activities complied with the principles of the Declaration of Istanbul.

### Statistical Analysis

Results are expressed as numbers (percentages) and means ± SD. The chi-square test or 1-way ANOVA was used to compare variables between groups. The *t* test was applied post hoc to compare specific pairs of groups when ANOVA indicated a significant difference. Allograft loss–free survival according to G1/G2 status was evaluated using Kaplan-Meier estimates, log-rank tests, and Cox proportional hazards models in both univariate and multivariate analyses. Survival time was calculated from the date of transplantation. The criterion used for censoring was the absence of the specified endpoint at the last follow-up. The last follow-up was defined as the date of the last follow-up at the kidney transplant clinic, in-person or via telemedicine. Statistical analyses were performed using Stata version 11.0 (StataCorp, College Station, TX) and SPSS version 30 (IBM, Chicago, IL). A *P* value of <0.05 was considered statistically significant.

## RESULTS

### Clinical Characteristics of the Cohort

The demographic and clinical characteristics of the cohort are summarized in Table 1. Among the 821 donor-recipient pairs, 251 (31%) donors were G1G1, 413 (50%) were G1G2, and 157 (19%) were G2G2. Baseline characteristics were comparable among the 3 groups. Donor-recipient class I HLA mismatches were also similar across groups. For HLA-DQB1, as expected—given that G1 encompasses a greater number of distinct genotypes than G2—we observed a higher number of mismatches in recipients of G1G1 donor grafts than in those receiving G2G2 grafts, as well as in recipients of G1G2 organs compared with those of G2G2 organs. An interleukin-2 receptor inhibitor was used as the induction agent in 86% of patients and antithymocyte globulin in 11%, with no significant difference between the groups. Initial maintenance immunosuppression was triple therapy in all patients, consisting of prednisone (100%), tacrolimus (99%) or cyclosporine (1%), and mycophenolate (99%) or azathioprine (1%). Only 1 patient in the cohort received rapamycin as initial therapy.

**TABLE 1.** Demographic and clinical characteristics of the cohort according to the donor HLA-DQ group genotype

	Donor G1G1 (N = 251)	Donor G1G2 (N = 413)	Donor G2G2 (N = 157)	*P*
Recipient				
Age at transplantation, y	51 ± 13	51 ± 15	53 ± 14	0.22
Male sex	151 (60)	251 (61)	91 (58)	0.83
Ethnicity				
American Indian	10 (4)	14 (3)	3 (2)	0.70
Asian	1 (0.4)	3 (1)	1 (1)	
Black	0 (0.0)	3 (1)	2 (1)	
Hispanic	1 (0.4)	1 (0.2)	0 (0)	
White	236 (94)	380 (92)	149 (95)	
Others	1 (0.4)	1 (0.2)	0 (0.0)	
Unknown	2 (1)	11 (3)	2 (1)	
Donor				
Age, y	49 ± 15	49 ± 15	49 ± 15	0.84
Type				
Deceased	185 (74)	328 (79)	120 (76)	0.23
HLA mismatch				
HLA-A	1.16 ± 0.68	1.20 ± 0.70	1.11 ± 0.68	0.32
HLA-B	1.34 ± 0.68	1.43 ± 0.68	1.34 ± 0.73	0.16
HLA-C	1.10 ± 0.68	1.05 ± 0.74	0.96 ± 0.70	0.13
HLA-DR	0.77 ± 0.66^*a*^	0.98 ± 0.71	0.85 ± 0.79	<0.01
HLA-DQB1	0.83 ± 0.72^*b*^	0.90 ± 0.70^*c*^	0.67 ± 0.75	0.01
0 MM	90 (36)	124 (30)	78 (50)	
1 MM	113 (45)	208 (50)	53 (34)	<0.01
2 MM	48 (19)	81 (20)	26 (17)	

Data are provided as mean ± SD or n (%). Comparisons were performed 1-way ANOVA or chi-square test.

^*a*^*P* < 0.01 vs G1G2.

^*b*^*P *= 0.03 vs G2G2.

^*c*^*P *< 0.01 vs G2G2.

CI, confidence interval; HR, hazard ratio.

### G1/G2 Status and Graft Failure

**FIGURE 1.**
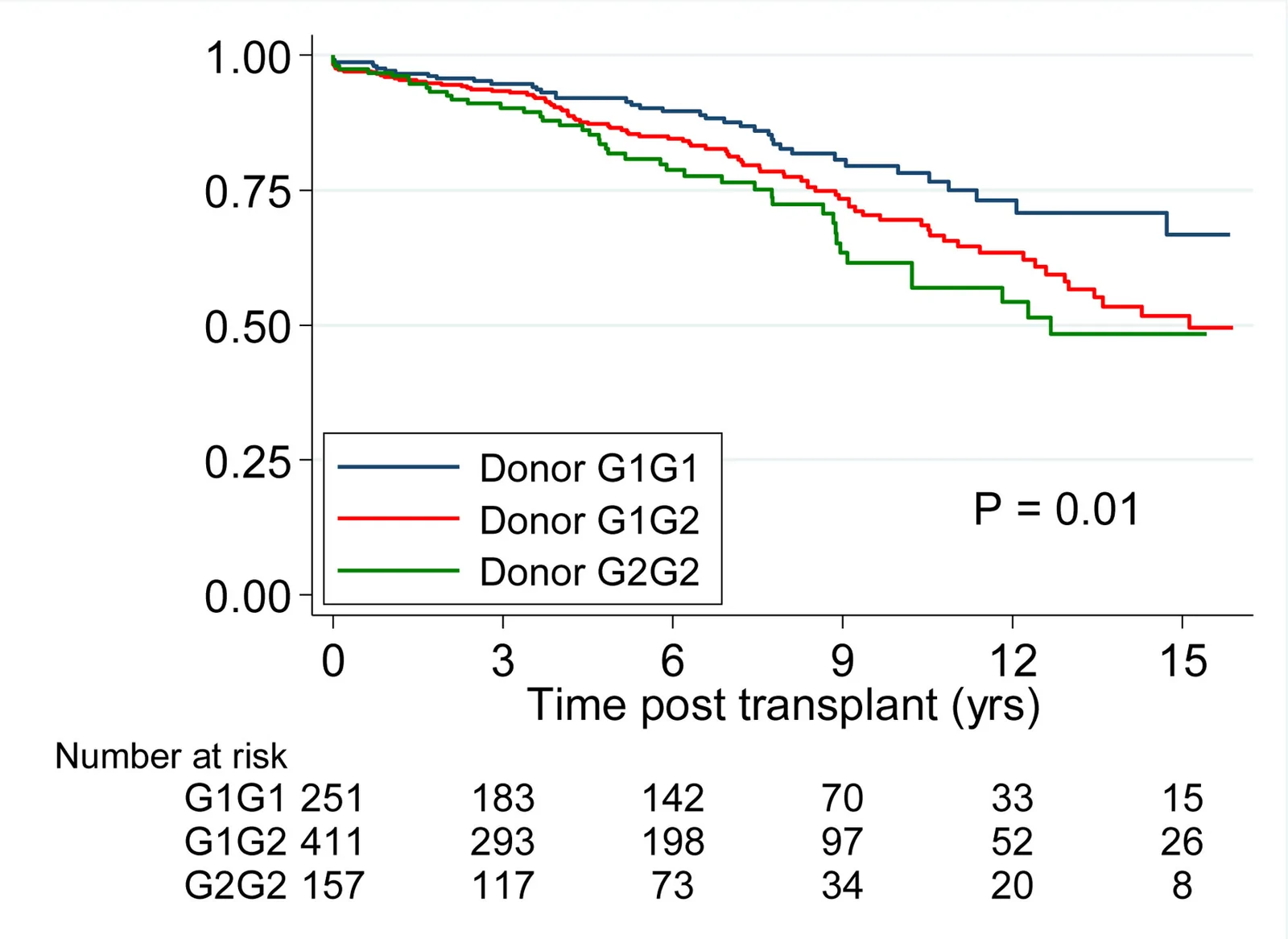
Kaplan-Meier plots of graft survival by donor HLA-DQ genotype. Comparison was performed using the log-rank test. G1, group 1 HLA-DQ heterodimer; G2, group 2 HLA-DQ heterodimer.

Overall, the median follow-up time after transplantation was 6.0 y (25th–75th percentiles: 2.4–9.0 y). During this period, 180 individuals (22%) experienced graft loss at a median of 5.2 y (interquartile range, 2.3–8.9 y). The proportion of graft loss during follow-up increased significantly from 16% to 23% and 28% among recipients of G1G1, G1G2, and G2G2 grafts, respectively (*P* = 0.01; Table 2, total graft failure). In survival analyses, recipients of G1G2 and G2G2 donor grafts had 1.6- and 1.9-fold higher risks of graft loss, respectively, compared with those receiving G1G1 grafts (Figure 1; Table 2, total graft failure). Multivariate modeling indicated that these associations were independent of the degree of HLA-DQ mismatch as well as of combined HLA-DQ and -DR mismatches. Analysis limited to death-censored graft loss showed similar risk estimates (**Table S1, SDC,** https://links.lww.com/TXD/A894). We then specifically evaluated graft failures due to biopsy-proven rejection, censoring other graft losses (Table 2, graft failure due to rejection). The biopsy diagnosing rejection was performed at a median (25–75th percentiles) of 2.1 (0.4–3.3) y before graft failure. The phenotypes were the following: mixed antibody-mediated rejection (AMR)/T cell–mediated rejection (TCMR) with acute and chronic components (29%), chronic TCMR (24%), chronic active AMR (19%), acute AMR (14%), and acute TCMR (14%). In this analysis, the risk was >4 times higher for G2G2 than for G1G1 kidneys.

**TABLE 2.** Risk estimates for graft failure associated with donor HLA-DQ heterodimer group

HLA-DQ group genotype	Events, n (%)	HR (95% CI; *P*)	Adjusted HR,^*a*^ (95% CI; *P*)	Adjusted HR,^*b*^ (95% CI; *P*)
Total graft failure				
G1G1	40/251 (16%)	Reference	Reference	Reference
G1G2	96/413 (23%)	1.6 (1.1-2.3; 0.01)	1.6 (1.1-2.3; 0.01)	1.6 (1.1-2.3; 0.02)
G2G2	44/157 (28%)	1.9 (1.2-2.9; <0.01)	1.9 (1.2-2.9; <0.01)	1.9 (1.2-2.9; <0.01)
Graft failure due to rejection				
G1G1	3/251 (1%)	Reference	Reference	Reference
G1G2	10/413 (2%)	2.2 (0.6-8.0; 0.23)	2.1 (0.6-7.8; 0.25)	2.1 (0.6-7.7; 0.26)
G2G2	8/157 (5%)	4.4 (1.2-16.8; 0.03)	4.4 (1.2-16.5; 0.03)	4.3 (1.1-16.4; 0.03)

Analysis was performed using Cox proportional hazards models.

^*a*^Adjusted for HLA-DQB1 mismatches.

^*b*^Adjusted for HLA-DQB1 and HLA-DR mismatches.

CI, confidence interval; HR, hazard ratio.

We next evaluated the impact of donor-recipient matching specifically within the G1/G2 groups. Patients were classified into the following mutually exclusive genotypic categories: (1) both donor and recipient with at least 1 G1 molecule (donor G1G1, no G1 mismatch; n = 237); (2) both donor and recipient with G1 and G2 molecules (donor/recipient G1G2, no G1G2 mismatch; n = 216); (3) both donor and recipient with at least a G2 molecule (Donor G2G2, no G2 mismatch; n = 133); (4) donor G1 mismatched (n = 87); and (5) donor G2 mismatched (n = 148). Compared with recipients of donors G1G1 without G1 mismatch, those who received G1G2 or G2G2 donors without mismatch had 1.6- and 1.8-fold higher risks of graft loss, respectively, whereas recipients of G1- or G2-mismatched kidneys had increased, albeit nonsignificant, risk of graft failure (Table 3).

**TABLE 3.** Risk estimates for graft failure associated with donor-recipient HLA-DQ heterodimer group match

HLA-DQ group genotype	Events, n (%)	HR (95% CI; *P*)	Adjusted HR,^*a*^ (95% CI; *P*)
Donor G1G1, no G1 MM	39/237 (17%)	Reference	Reference
Donor/recipient G1G2, no G1G2 MM	51/216 (24%)	1.5 (1.0-2.3; 0.04)	1.6 (1.0-2.4; 0.03)
Donor G2G2, no G2 MM	38/133 (29%)	1.8 (1.2-2.8; 0.01)	1.8 (1.2-2.9; 0.01)
Donor/recipient G1 MM^*b*^	20/87 (23%)	1.8 (1.0-3.0; 0.04)	1.7 (0.97-2.9; 0.07)
Donor/recipient G2 MM^*c*^	32/148 (22%)	1.6 (1.0-2.6; 0.045)	1.5 (0.9-2.5; 0.10)

Analysis was performed using Cox proportional hazards models.

^*a*^Adjusted for HLA-DQB1 and HLA-DR mismatches.

^*b*^Only donor with G1 molecule.

^*c*^Only donor with G2 molecule.

CI, confidence interval; HR, hazard ratio.

Taken together, these results suggest that the adverse clinical impact associated with the G2 genotype reported in bone marrow transplant recipients is consistently observed in kidney transplantation.

### Donor Serologic Antigen Family DQ1, DQ3, and Graft Failure

Given our previously reported relationship between HLA-DQB genotypes and cell surface HLA protein levels—with HLA-DQ5 and -DQ6 (further referred to as the HLA-DQ1 group) exhibiting the highest and HLA-DQ7, -DQ8, and -DQ9 (further referred to as the HLA-DQ3 group) the lowest expression levels^7^—we investigated whether the impact of donor G1/G2 status on outcomes could be refined by examining these specific genotypes. The HLA-DQ1 group corresponds exclusively to G2, whereas the HLA-DQ3 group represents only a subset of G1, which also includes HLA-DQ2 and HLA-DQ4.

To address this question, we classified patients into the following mutually exclusive donor genotypic categories, as described previously^7^: (1) at least 1 DQ3 allele and no DQ1 allele (DQ3^+^DQ1^–^; reference group), (2) 1 DQ3 allele and 1 DQ1 allele (DQ3^+^DQ1^+^), (3) no DQ3 allele and 1 DQ1 allele (DQ3^–^DQ1^+^), and (4) neither DQ1 nor DQ3 allele (DQ3^–^DQ1^–^). Given the underlying biology of the G1/G2 groupings, this last DQ3^–^DQ1^–^ category belonged to G1, with homozygous or heterozygous genotypes for HLA-DQ2 and HLA-DQ4.

First, compared with recipients of DQ3^+^DQ1^–^ donor grafts, recipients of DQ3^–^Q1^+^ donor grafts had a 2.1-fold higher risk of graft loss (Table 4, top). Second, recipients of heterozygous DQ3^+^DQ1^+^ donor grafts also had poorer outcomes than those receiving DQ3^+^DQ1^–^ grafts. Third, recipients of DQ3^-^DQ1^-^ donor grafts had a similar risk to that observed among recipients of DQ3^+^DQ1^+^ donor grafts. In other words, the protective effect noted earlier for the G1G1 group appeared to be confined to the HLA-DQ3 donor subgroup. Analysis limited to death-censored graft loss showed similar risk estimates (**Table S2, SDC,** https://links.lww.com/TXD/A894). Specifically, compared with recipients of DQ3^+^DQ1^–^ donor grafts, recipients of DQ3^–^DQ1^+^ donor grafts had a 2.7-fold higher risk of death-censored graft loss (**Table S2, SDC,** https://links.lww.com/TXD/A894). In addition, recipients of DQ3^–^DQ1^–^ donor grafts had a hazard ratio of 2.5 for death-censored graft failure, compared with 1.5 among recipients of DQ3^+^DQ1^+^ donor grafts (**Table S2, SDC,** https://links.lww.com/TXD/A894). When only biopsy-proven rejection losses were considered, the risk was >4 times higher for DQ3^–^DQ1^+^ donor kidneys compared with DQ3^+^DQ1^–^ donors (hazard ratio, 4.7; 95% confidence interval, 1.1-20.7; *P* = 0.04; **Table S3, SDC,** https://links.lww.com/TXD/A894).

**TABLE 4.** Risk estimates for graft failure associated with donor HLA-DQ1 and -DQ3 genotype

HLA-DQ genotype	Events, n (%)	HR (95% CI; *P*)	Adjusted HR,^*a*^ (95% CI; *P*)
DQ3^+^DQ1^–^	31/210 (15%)	Reference	Reference
DQ3^+^DQ1^+^	49/219 (22%)	1.6 (1.0-2.5; 0.05)	1.6 (1.01-2.5; 0.046)
DQ3^–^DQ1^+^	91/351 (26%)	2.0 (1.4-3.1; <0.01)	2.1 (1.4-3.1; <0.01)
DQ3^–^DQ1^–^	9/41 (22%)	1.8 (0.8-3.6; 0.15)	1.8 (0.9-3.8; 0.12)
DQ3^++^DQ1^–^	14/106 (13%)	Reference	Reference
DQ3^+^DQ1^–^	17/104 (16%)	1.3 (0.6-2.6; 0.51)	1.3 (0.6-2.7; 0.44)
DQ3^+^DQ1^+^	49/219 (22%)	1.8 (0.98-3.2; 0.06)	1.9 (1.0-3.4; 0.049)
DQ3^–^DQ1^+^	47/194 (24%)	2.3 (1.3-4.2; 0.01)	2.4 (1.3-4.4; 0.01)
DQ3^–^DQ1^++^	44/157 (28%)	2.3 (1.3-4.2; 0.01)	2.5 (1.3-4.5; 0.01)
DQ3^–^DQ1^–^	9/41 (22%)	1.9 (0.9-4.5; 0.12)	2.1 (0.9-4.5; 0.09)

Analysis was performed using Cox proportional hazards models.

^*a*^Adjusted for HLA-DQB1 and HLA-DR mismatches.

CI, confidence interval; HR, hazard ratio.

To better understand these associations, we further analyzed the association between HLA-DQ1 and HLA-DQ3 genotypes and graft loss, considering the number of alleles in each genotype. In absolute terms, recipients of donor grafts carrying 2 HLA-DQ1 alleles (DQ3^–^DQ1^++^) had more than double the incidence of graft loss compared with those receiving grafts from donors carrying 2 HLA-DQ3 alleles (Table 4, bottom). Multivariate survival modeling, adjusted for HLA-DQB1 and -DR mismatches, confirmed a 2.5-fold higher risk of graft loss in recipients of HLA-DQ1-homozygous donor grafts.

Taken together, these findings suggest that the beneficial impact of donor G1 on graft outcomes is primarily attributable to the HLA-DQ3 allele, with a gradient of protection corresponding to the number of HLA-DQ3 alleles.

### Sensitivity Analyses

To be a confounder, a factor must possess the following characteristics: it must be associated with the outcome, it must be associated with the exposure factor, and it must not be an effect of the exposure (it must not be in the causal pathway between the exposure and the outcome).^19^ In the present case, the HLA genotype is not affected by the various transplant eras. Similarly, there is no association between the HLA genotype and basic characteristics such as age, sex, and donor type. Nevertheless, we conducted a sensitivity analysis in which we adjusted for all factors in Table 1, in addition to the year posttransplant, cold ischemia time, delayed graft function, induction therapy, and baseline maintenance immunosuppression. This analysis confirmed a significant risk associated with the donor genotypes G1G2 and G2G2 (**Table S4, SDC,** https://links.lww.com/TXD/A894). It also confirmed that recipients of G2G2 kidneys without mismatch in G2 had an increased risk compared with recipients of G1G1 kidneys without mismatch (**Table S5, SDC,** https://links.lww.com/TXD/A894). In addition, the graft survival analyses according to the HLA-DQ1 and -DQ3 alleles were robust to multivariable adjustment for all associations demonstrated in the primary analysis (**Table S6, SDC,** https://links.lww.com/TXD/A894).

Finally, we analyzed the association between G1/G2 donor status and the development of dnDSA. A total of 23 patients (2.8%) developed dnDSA, at a median of 3.0 y (interquartile range, 0.6–5.5 y). Among these, 14 patients had dnDSA against HLA-DQ. The proportion of recipients of G1G2 donors who developed dnDSA was higher than that of G1G1 recipients; this association did not appear to be confounded (**Table S7, SDC,** https://links.lww.com/TXD/A894). When the analysis focused on HLA-DQ dnDSA, we observed no cases among recipients of G1G1 donors, which prevented analysis using the Cox model; however, there was a statistical difference between groups when compared using the log-rank test (*P* = 0.03). Overall, this analysis supports the notion that recipients of G1G1 donors appeared at lower risk of alloimmune events.

## DISCUSSION

The relevance of HLA-DQ mismatching in transplantation outcomes is well established. Historically, graft compatibility assessment and the detection of anti-HLA antibodies have been limited to evaluating HLA mismatches. This necessary clinical simplification of a complex biological system has likely hindered a full understanding of underlying immunological mechanisms. The primary objective of this study was to determine whether recent observations regarding G1/G2 HLA-DQαβ heterodimer status in hematopoietic cell transplantation also apply to solid organ transplantation. Our findings demonstrate that the risk of graft loss was higher among recipients of G1G2 and G2G2 grafts than among those receiving G1G1 grafts. Multivariable analyses further revealed that these associations were independent of recipient G1/G2 status, HLA-DR mismatches, and HLA-DQB1 mismatches.

Related to the search for an underlying mechanism, the second objective was to compare the risk of allograft loss according to HLA-DQ1 and -DQ3 genotypes. The rationale for this analysis stemmed from our previous observation that HLA-DQ1 alleles are associated with high endothelial surface expression of HLA-DQ molecules, whereas HLA-DQ3 alleles are associated with lower expression levels, potentially influencing immune responses.^7^ In the current study, we found that HLA-DQ1 (DQ5 and DQ6) alleles were associated with increased risks of graft loss compared with HLA-DQ3 alleles. Furthermore, HLA-DQ2 and HLA-DQ4—other alleles forming part of G1 along with HLA-DQ3—were also associated with similarly increased risks, comparable with those of HLA-DQ1. These findings refine the previously proposed G1/G2 risk paradigm, suggesting that the protective effect attributed to G1 may, in fact, be restricted to the subgroup of HLA-DQ3 alleles. Consistent with this interpretation, the presence of 2 HLA-DQ3 alleles conferred greater protection than a single allele for graft loss.

On the same topic, but posing a different question, Maguire et al^10^ recently reported, in a subset of the US Scientific Registry of Transplant Recipients—2922 recipients of a G2G2 kidney—that death-censored graft survival was better when the recipient was homozygous G2G2, compared with heterozygous G1G2 or homozygous G1G1 recipients. As expected, these data showed that HLA matching, in this case at the G2 level, confers better graft survival. These results are not inconsistent with those presented here, because they did not analyze the impact of G1 versus G2 molecules but focused on the HLA match in a subcohort of G2G2 kidney recipients.

Overall, these findings further underscore the limitations in our current understanding of the immunological role of HLA-DQ antigens. In vitro transduction studies examining cell-surface HLA expression among HLA-DQ alleles have demonstrated substantial variation in surface protein levels, consistent with our previous observations in endothelial colony-forming cells generated from human peripheral blood mononuclear cell samples.^7,20^ Using immortalized mouse fibroblasts (NIH3T3 cells) transduced with various HLA-DQA1 and DQB1 genes, Miyadera et al^20^ reported that protein expression levels differed according to heterodimeric combinations. Although they did not specifically use the G1/G2 terminology, their data indicated that surface protein levels were generally lower for G1 than for G2 molecules. They proposed that the higher risk of type 1 diabetes associated with HLA-DQB1*03:02 (commonly known as HLA-DQ8) could be attributed to lower surface protein levels, which may impair thymic deletion of autoreactive CD4^+^ T cells during immune development.

From a transplantation perspective, it is tempting to speculate that the biologically relevant mechanism underlying poorer clinical outcomes involves the higher density of HLA-DQ molecules in G2 individuals, which could enhance both alloreactive and nonalloreactive responses. Our subanalysis of graft loss due to rejection supports the idea that increased alloreactivity is at play. It can be speculated that higher expression of G2 molecules increases not only the risk of rejection episodes but also the risk of graft failure and mortality from infection and cancer because of the subsequent increase in immunosuppression. A large, multicenter cohort will be necessary to robustly test such immunological hypotheses.

The risk of graft loss we observed in recipients of G2G2 grafts was 1.9 times higher than in recipients of G1G1 grafts. In comparison, the study published by Port et al^21^ in 2002 on donor characteristics associated with reduced graft survival, which led to the extended criteria donor, showed a relative risk of 1.7. This study did not lead to a discontinuation of extended criteria donor kidneys, but it did prompt significant changes in practice, depending on the country and transplant center. Additional external data will be needed to verify the generalizability of the results obtained here, but external confirmation would have multiple implications. Clinically, the data would provide a strong incentive to consider HLA-DQ genotyping in donor risk assessment. Confirmation of the increased risk of G2G2 graft loss due to rejection would also be an incentive for increased posttransplant surveillance, either through screening for anti-HLA antibodies or lower clinical thresholds for performing a biopsy. From a biological perspective, it would generate a strong need to understand the underlying immunological mechanisms. Research exploring differential immunogenicity by genotype, particularly with respect to HLA eplets, is rapidly advancing.^8,22-24^ A major challenge in translating these in silico findings into practice lies in determining their true functional impact on immune responses.^10,25,26^ The present results strengthen the concept that HLA genetic variability influences transplant outcomes beyond classical alloreactivity driven by donor-recipient mismatches. Concurrent analyses of surface protein expression, genotype, and clinical outcomes will be required to elucidate the underlying mechanisms.

This study has limitations. First, it involved a relatively homogeneous cohort composed primarily of White participants. Although this does not invalidate the findings, it limits their generalizability. Second, despite the long follow-up period, the number of events was small in some subgroups. A larger multicenter cohort would enable testing whether specific causes of graft failure are associated with heterodimer groups or specific alleles. Such analyses, however, may be subject to survival bias, as patients at lower risk of graft loss due to alloreactivity have longer exposure to adverse effects of immunosuppression. Determining DSA by association when DQA1 typing was unknown, as well as the retrospective analysis of rejection diagnoses, are 2 limitations that may have resulted in a different number of patients with graft loss due to rejection than would have been obtained with a more contemporary analysis or cohort. However, given that the various Banff criteria represent an expert consensus that evolves over time, not only in the past but also in the future, using the criteria in effect at the time the grafts were lost does not invalidate the analysis itself.

In summary, the data presented here suggest an association between the G2 group of HLA-DQ heterodimers and kidney transplant failure, independent of HLA-DR and HLA-DQ mismatches. They further suggest that among G1 donors, those carrying the HLA-DQ3 genotype are associated with more favorable outcomes. These findings provide additional impetus to elucidate the specific biological roles of the different classes of HLA molecules in transplantation.

## ACKNOWLEDGMENTS

The authors thank all individuals who participated in this study. This work was supported by the Kidney Foundation of Canada through Research Grant 24KHRG-1253388 and Canadian Institutes of Health Research PJT-166018. S.A.D.S. received a scholarship from the Fonds de Recherche du Québec–Santé (FRQS 282015). M.P.T. received scholarships from the Fondation du CHU de Québec.

## Supplementary Material


