# Supplementary material for: Association Between Donor HLA-DQ Heterodimers Group and Graft Loss in Kidney Transplant Recipients
Source: Transplant Direct. 2026 Sep 9;12(10):e1998. doi: 10.1097/TXD.0000000000001998 (PMC13561284; doi:10.1097/TXD.0000000000001998)
Supplement: Supplementary file 1 [file txd-12-e1998-s001.pdf]

SUPPLEMENTAL DIGITAL CONTENT to Thivierge MP *et al.*

SUPPLEMENTAL TABLES

Table S1. Risk estimates for death-censored graft failure associated with donor HLA-DQ heterodimer group

HLA-DQ group genotype	Events n (%)	HR (95% CI; P)	Adjusted HR[1] (95% CI; P)	Adjusted HR[2] (95% CI; P)
G1G1	14/251 (6%)	ref.	ref.	ref.
G1G2	37/413 (9%)	1.7 (0.9-3.2; 0.08)	1.7 (0.9-3.1; 0.10)	1.6 (0.8-3.0; 0.16)
G2G2	17/157 (11%)	2.0 (1.0-4.1; 0.05)	2.0 (1.0-4.2; 0.05)	1.9 (0.9-4.0; 0.08)

Analysis was performed using Cox proportional hazards models. HR, hazard ratio.

[1] adjusted for HLA-DQB1 and HLA-DR mismatches

[2] adjusted for HLA-DQB1 mismatches, HLA-DR mismatches, recipient age at transplant, HLA A, B, and C mismatch, sex, ethnicity, donor age, donor type, year of transplant, cold ischemia time, delayed graft function, induction therapy, baseline immunosuppression (prednisone, tacrolimus/cyclosporine, mycophenolate, azathioprine, rapamycin)

Table S2. Risk estimates for death-censored graft failure associated with donor HLA-DQ1 and -DQ3 genotype

HLA-DQ genotype	Events n (%)	HR (95% CI; P)	Adjusted HR[1] (95% CI; P)
$DQ3^{+}$ $DQ1^{-}$	10/210 (5%)	ref.	ref.
$DQ3^{+}$ $DQ1^{+}$	15/219 (7%)	1.5 (0.7-3.3; 0.33)	1.5 (0.7-3.3; 0.34)
$DQ3^{-}$ $DQ1^{+}$	39/351 (11%)	2.6 (1.3-5.3; 0.01)	2.7 (1.3-5.4; 0.01)
$DQ3^{-}$ $DQ1^{-}$	4/41 (10%)	2.4 (0.7-7.6; 0.15)	2.5 (0.8-8.1; 0.12)
$DQ3^{++}$ $DQ1^{-}$	5/106 (5%)	ref.	ref.
$DQ3^{+}$ $DQ1^{-}$	5/104 (5%)	1.1 (0.3-3.7; 0.91)	1.0 (0.3-3.5; 0.44)
$DQ3^{+}$ $DQ1^{+}$	15/219 (7%)	1.85 (0.6-4.2; 0.40)	1.5 (0.5-4.1; 0.049)
$DQ3^{-}$ $DQ1^{+}$	22/194 (11%)	2.9 (1.1-7.7; 0.03)	2.8 (1.1-7.5; 0.04)
$DQ3^{-}$ $DQ1^{++}$	17/157 (11%)	2.5 (0.9-6.9; 0.07)	2.5 (0.9-6.9; 0.07)
$DQ3^{-}$ $DQ1^{-}$	4/41 (10%)	2.5 (0.7-9.2; 0.18)	2.5 (0.7-1.5; 0.91)

Analysis was performed using Cox proportional hazards models. HR, hazard ratio.

[1] adjusted for HLA-DQB1 and HLA-DR mismatches

Table S3. Risk estimates for graft failure due to rejection associated with donor HLA-DQ1 and -DQ3 genotype

HLA-DQ genotype	Events n (%)	HR (95% CI; P)	Adjusted HR[1] (95% CI; P)
$DQ3^+ DQ1^-$	2/210 (1%)	ref.	ref.
$DQ3^+ DQ1^+$	4/212 (2%)	2.0 (0.4-11.0; 0.42)	2.0 (0.4-10.8; 0.44)
$DQ3^- DQ1^+$	14/358 (4%)	4.8 (1.1-21.1; 0.04)	4.7 (1.1-20.7; 0.04)
$DQ3^- DQ1^-$	1/41 (2%)	3.2 (0.3-35.1; 0.35)	3.2 (0.3-35.8; 0.35)

Analysis was performed using Cox proportional hazards models. HR, hazard ratio.

[1] adjusted for HLA-DQB1 and HLA-DR mismatches

Table S4. Risk estimates for graft failure associated with donor HLA-DQ heterodimer group

HLA-DQ group genotype	Events n (%)	HR (95% CI; P)	Adjusted HR[1] (95% CI; P)	Adjusted HR[2] (95% CI; P)
Total graft failure				
G1G1	40/251 (16%)	ref.	ref.	ref.
G1G2	96/413 (23%)	1.6 (1.1-2.3; 0.01)	1.6 (1.1-2.3; 0.02)	1.6 (1.1-2.4; 0.02)
G2G2	44/157 (28%)	1.9 (1.2-2.9; <0.01)	1.9 (1.2-2.9; <0.01)	1.8 (1.1-2.8; 0.02)
Graft failure due to rejection				
G1G1	3/251 (1%)	ref.	ref.	ref.
G1G2	10/413 (2%)	2.2 (0.6-8.0; 0.23)	2.1 (0.6-7.7; 0.25)	1.4 (0.3-5.7; 0.67)
G2G2	8/157 (5%)	4.4 (1.2-16.8; 0.03)	4.3 (1.1-16.4; 0.03)	4.6 (1.1-20.1; 0.04)

Analysis was performed using Cox proportional hazards models. HR, hazard ratio.

[1] adjusted for HLA-DQB1 and HLA-DR mismatches

[2] adjusted for HLA-DQB1 mismatches, HLA-DR mismatches, recipient age at transplant, HLA A, B, and C mismatch, sex, ethnicity, donor age, donor type, year of transplant, cold ischemia time, delayed graft function, induction therapy, baseline immunosuppression (prednisone, tacrolimus/cyclosporine, mycophenolate, azathioprine, rapamycin)

Table S5. Risk estimates for graft failure associated with donor/recipient HLA-DQ heterodimer group match

HLA-DQ group genotype	Events n (%)	HR[3] (95% CI; P)	Adjusted HR[4] (95% CI; P)
Donor G1G1, no G1 MM	39/237 (17%)	ref.	ref.
Donor/recipient G1G2, no G1G2 MM	51/216 (24%)	1.6 (1.0-2.4; 0.03)	1.6 (1.01-2.4; 0.046)
Donor G2G2, no G2 MM	38/133 (29%)	1.8 (1.2-2.9; 0.01)	1.7 (1.064-2.8; 0.03)
Donor/recipient G1 MM[1]	20/87 (23%)	1.7 (0.97-2.9; 0.07)	1.8 (0.98-3.2; 0.06)
Donor/recipient G2 MM[2]	32/148 (22%)	1.5 (0.9-2.5; 0.10)	1.5 (0.9-2.6; 0.10)

Analysis was performed using Cox proportional hazards models. HR, hazard ratio.

[1] Only donor with G1 molecule

[2] Only donor with G2 molecule

[3] adjusted for HLA-DQB1 and HLA-DR mismatches

[4] adjusted for HLA-DQB1 mismatches, HLA-DR mismatches, recipient age at transplant, HLA A, B, and C mismatch, sex, ethnicity, donor age, donor type, year of transplant, cold ischemia time, delayed graft function, induction therapy, baseline immunosuppression (prednisone, tacrolimus/cyclosporine, mycophenolate, azathioprine, rapamycin)

Table S6. Risk estimates for graft failure associated with donor HLA-DQ1 and -DQ3 genotype

HLA-DQ genotype	Events n (%)	HR[1] (95% CI; P)	Adjusted HR[2] (95% CI; P)
$DQ3^{+}$ $DQ1^{-}$	31/210 (15%)	ref.	ref.
$DQ3^{+}$ $DQ1^{+}$	49/219 (22%)	1.6 (1.01-2.5; 0.046)	1.7 (1.01-2.7; 0.03)
$DQ3^{-}$ $DQ1^{+}$	91/351 (26%)	2.1 (1.4-3.1; <0.01)	1.9 (1.2-2.9; <0.01)
$DQ3^{-}$ $DQ1^{-}$	9/41 (22%)	1.8 (0.9-3.8; 0.12)	1.7 (0.8-3.7; 0.22)
$DQ3^{++}$ $DQ1^{-}$	14/106 (13%)	ref.	ref.
$DQ3^{+}$ $DQ1^{-}$	17/104 (16%)	1.3 (0.6-2.7; 0.44)	1.4 (0.7-3.0; 0.33)
$DQ3^{+}$ $DQ1^{+}$	49/219 (22%)	1.9 (1.0-3.4; 0.049)	2.0 (1.1-3.8; 0.03)
$DQ3^{-}$ $DQ1^{+}$	47/194 (24%)	2.4 (1.3-4.4; 0.01)	2.3 (1.2-4.3; 0.01)
$DQ3^{-}$ $DQ1^{++}$	44/157 (28%)	2.5 (1.3-4.5; 0.01)	2.3 (1.2-4.3; 0.01)
$DQ3^{-}$ $DQ1^{-}$	9/41 (22%)	2.1 (0.9-4.5; 0.09)	2.1 (0.8-5.1; 0.11)

Analysis was performed using Cox proportional hazards models. HR, hazard ratio.

[1] adjusted for HLA-DQB1 and HLA-DR mismatches

[2] adjusted for HLA-DQB1 mismatches, HLA-DR mismatches, recipient age at transplant, HLA A, B, and C mismatch, sex, ethnicity, donor age, donor type, year of transplant, cold ischemia time, delayed graft function, induction therapy, baseline immunosuppression (prednisone, tacrolimus/cyclosporine, mycophenolate, azathioprine, rapamycin)

Table S7. Risk estimates for dnDSA associated with donor HLA-DQ heterodimer group

HLA-DQ group genotype	Events n (%)	HR (95% CI; P)	Adjusted HR[1] (95% CI; P)	Adjusted HR[2] (95% CI; P)
Any DSA				
G1G1	1/251 (0.4%)	ref.	ref.	ref.
G1G2	18/413 (4%)	11.8 (1.5-88; 0.02)	10.6 (1.4-79; 0.02)	11.7 (1.5-92; 0.02)
G2G2	4/157 (2.5%)	6.7 (0.8-59; 0.09)	6.4 (0.7-58; 0.10)	6.6 (0.7-62; 0.10)
HLA-DQ dnDSA				
G1G1	0/251 (0%)	N/A[3]	N/A[3]	N/A[3]
G1G2	11/413 (2.7%)	N/A	N/A	N/A
G2G2	3/157 (1.9%)	N/A	N/A)	N/A

Analysis was performed using Cox proportional hazards models. HR, hazard ratio; dn DSA, *de novo* donor-specific antibody.

1 adjusted for HLA-DQB1 and HLA-DR mismatches

2 adjusted for HLA-DQB1 mismatches, HLA-DR mismatches, recipient age at transplant, HLA A, B, and C mismatch, sex, ethnicity, donor age, donor type, year of transplant, cold ischemia time, delayed graft function, induction therapy, baseline immunosuppression (prednisone, tacrolimus/cyclosporine, mycophenolate, azathioprine, rapamycin)

3 Cox models could not be computed due to the absence of event in the G1G1 group.
